# Influence of Glucose on *Candida albicans* and the Relevance of the Complement FH-Binding Molecule Hgt1 in a Murine Model of Candidiasis

**DOI:** 10.3390/antibiotics11020257

**Published:** 2022-02-16

**Authors:** Verena Harpf, Samyr Kenno, Günter Rambach, Verena Fleischer, Nadia Parth, Christian X. Weichenberger, Peter Garred, Silke Huber, Cornelia Lass-Flörl, Cornelia Speth, Reinhard Würzner

**Affiliations:** 1 Institute of Hygiene and Medical Microbiology, Medical University of Innsbruck, 6020 Innsbruck, Austria; verena.harpf@i-med.ac.at (V.H.); samyr.kenno@uksh.de (S.K.); guenter.rambach@i-med.ac.at (G.R.); verena.fleischer@i-med.ac.at (V.F.); nadia.parth@i-med.ac.at (N.P.); silke.huber@i-med.ac.at (S.H.); cornelia.lass-floerl@i-med.ac.at (C.L.-F.); cornelia.speth@i-med.ac.at (C.S.); 2 Institute for Biomedicine (Affiliated to the University of Lübeck), Eurac Research, 39100 Bolzano, Italy; christian.weichenberger@eurac.edu; 3 Laboratory of Molecular Medicine, Department of Clinical Immunology Section 7631, Rigshospitalet, Copenhagen University Hospital, 2200 Copenhagen N, Denmark; peter.garred@regionh.dk

**Keywords:** *Candida albicans*, Hgt1, diabetes, complement, factor H, C3, murine model

## Abstract

Candidiasis is common in diabetic patients. Complement evasion is facilitated by binding complement factor H (FH). Since the expression of high-affinity glucose transporter 1 (Hgt1), a FH-binding molecule, is glucose-dependent, we aimed to study its relevance to the pathogenesis of *Candida albicans*. Euglycemic and diabetic mice were intravenously challenged with either *Candida albicans* lacking Hgt1 (hgt1-/-) or its parental strain (SN152). Survival and clinical status were monitored over 14 days. In vitro, *Candida albicans* strains were grown at different glucose concentrations, opsonized with human serum, and checked for C3b/iC3b and FH deposition. Phagocytosis was studied by fluorescein isothiocyanate-labeled opsonized yeast cells incubated with granulocytes. The murine model demonstrated a significantly higher virulence of SN152 in diabetic mice and an overall increased lethality of mice challenged with hgt1-/-. In vitro lower phagocytosis and C3b/iC3b deposition and higher FH deposition were demonstrated for SN152 incubated at higher glucose concentrations, while there was no difference on hgt1-/- at physiological glucose concentrations. Despite C3b/iC3b and FH deposition being glucose-dependent, this effect has a minor influence on phagocytosis. The absence of Hgt1 is diminishing this dependency on complement deposition, but it cannot be attributed to being beneficial in a murine model.

## 1. Introduction

The complement system plays a central role in innate immunity, bridging the innate and the adaptive immune defense [1]. This complex system consists of over 40 proteins, soluble plasma factors, cell-associated regulator molecules, and receptors [2]. After an appropriate surface-bound or soluble pattern recognition receptor gets in contact with either pathogen-associated molecular patterns or foreign cellular structures such as cell debris or non-self-tissue, the activation starts within seconds. This activation can take place by three pathways, the classical (CP), the lectin (LP), and the alternative pathway (AP). Those three activation pathways merge in the enzymatic activation of C3, and subsequent cleavage of C5 [1]. The cleavage product C5b exhibits a binding site for C6, starting the formation of the terminal complement complex. The nonenzymatically sequentially activated C7, C8, and C9 adhere to C5b6 to form this complex, which can either exist in soluble form or produce a lytic pore as a membrane–attack complex (MAC) [1].

To avoid potentially harmful inflammation and adaptive immune response, *Candida albicans*, which is normally a commensal of the human body, has evolved several immune evasion strategies [3,4,5,6,7,8,9]. The complement evasion strategies can be divided into three patterns; firstly, the masking from recognition by the complement, secondly, cleavage and blocking of complement proteins, and thirdly, recruitment of complement regulators. The β-glucan layer of *Candida albicans* is masked by a sheath of mannan, therefore inhibiting the recognition by the alternative pathway [3]. The essential characters of the second pattern of the complement evasion are secreted aspartyl proteases (Saps) [4]. For the third, besides the masking from complement and the destruction or blockage of various complement proteins, *Candida albicans* recruits proteins, which normally regulate the complement system to avoid detrimental effects on endogenous cells. Although bound to either yeast cells or pseudohyphae, those regulators keep their functions. Factor H (FH), the major regulator of the AP, and its truncated form, factor-H-like protein 1 (FHL-1), are acquired by *Candida albicans* using four different proteins, namely phosphoglycerate mutase 1 (Gpm1) [5], pH-regulated antigen 1 (Pra1) [6], high affinity glucose transporter 1 (Hgt1) [7], and glycerol-3-phosphate dehydrogenase 2 (Gpd2) [8]. Bound FH exerts three effector functions; the protein competes with factor B (FB) for the binding of C3b and therefore inhibits the assembly of the AP C3- and C5-convertases [9]. Furthermore, FH accelerates the decay of the C3-and C5-convertases by displacing bound factor Bb. Lastly, the acquisition of this regulator leads to ameliorated factor I (FI)-mediated cleavage and inactivation of C3b, preventing its participation in the terminal complement pathway [2]. As an opportunistic pathogen, this species can cause mucosal and disseminated infections under certain circumstances such as iatrogenic immunosuppression, transplantation, antibiotic treatment, and untreated diabetes mellitus [10], providing the pathogen a huge basis to spread.

In particular, diabetes mellitus is a metabolic disorder characterized by elevated blood sugar; fasting (8 h without food intake) plasma glucose levels of ≥126 mg/dL, or the 2-h plasma glucose level of ≥200 mg/dL [11], affect an estimated 463 million people in 2019 with increasing numbers due to sedentary living and high-energy dietary intake [12], especially type 2 diabetes [11]. Diabetes is linked to cardiovascular disease and alterations of the immune system, especially the cell-mediated immunity, affecting adherence, chemotaxis, phagocytosis, oxidative burst, and intracellular killing, which generates a higher susceptibility of diabetic patients to fungal and bacterial infections compared to euglycemic individuals [13]. Levels just below diabetic individuals are called prediabetes, which is linked to a higher risk of developing diabetes and cardiovascular disease [11].

Considering the higher susceptibility of diabetic patients to candidiasis and the glucose-dependent expression of the *Candida albicans* FH-binding molecule Hgt1, this study aimed to enlighten the relevance of this molecule, hyperglycemia, and the complement system on the pathogenesis of *Candida albicans*. 

## 2. Results

### 2.1. Absence of Hgt1 or C3 Increases the Virulence of Candida albicans in a Murine Model of Systemic Candidiasis

Preceding research showed the relevance of Hgt1 in complement evasion of *Candida albicans* [7]. Furthermore, it was found that the expression is glucose-dependent [14]. To visualize the importance of this protein in virulence, the *HGT1* knockout mutant hgt1-/- and the parental strain SN152 were studied in a murine model of disseminated candidiasis. 

**Figure 1:**
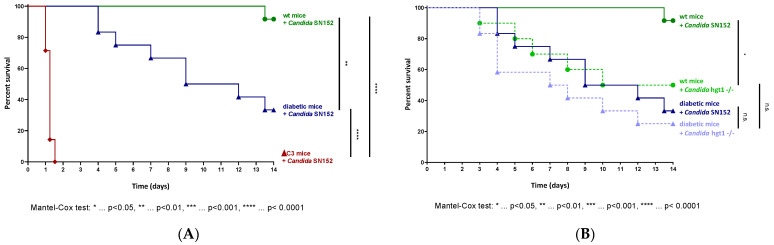
Survival curves in a murine model of candidemia. Euglycemic (wt, *n* = 12), diabetic (*n* = 12), and C3 deficient (ΔC3, *n* = 7) animals were intravenously injected with 6 × 10^2^ *Candida albicans* cells of the parental strain SN152 per gram mouse (**A**). Survival curves of wt (n*_Candida_* _SN152_ = 12, n*_Candida_* _hgt1-/-_ = 8) and diabetic animals (*n* = 12) challenged with 6 × 10^2^ yeast cells of either *Candida albicans* SN152 (―) or the *HGT1* knockout mutant (hgt1-/-, ----) per gram mouse were compared (**B**). The survival was monitored for 14 days. Stars (*) indicate the level of significance for a *p*-value resulting from a Mantel–Cox test analyzing pairs of survival curves.

When euglycemic mice were intravenously injected with 6 × 10^2^ yeast cells of the parental strain per gram mouse, this *Candida albicans* strain showed minimal virulence (Figure 1A). Diabetic animals infected with the same amount of yeast cells presented a significantly aggravating course of infection resulting in having to sacrifice 66.7% of the animals used for the experiment within 14 days (Figure 1A). SN152 induced the highest lethality in C3 deficient mice, in which the intravenous challenge led to a lethal outcome in all infected animals within two days (Figure 1A). 

Although it was revealed by us that Hgt1 is a complement evasion molecule [7], knocking out *HGT1* surprisingly led to a significantly higher virulence of *Candida albicans* compared to SN152 in euglycemic mice (Figure 1B; χ^2^(1) = 5.306, *p* = 0.0213). In diabetic animals, the same tendency was observed, but without a significant difference in virulence (Figure 1B). In contrast to SN152, challenging diabetic animals with 6 × 10^2^ yeast cells per gram mouse of the hgt1-/- did not lead to a significantly higher lethality compared to euglycemic mice infected with the same amount of yeast cells (Figure 1B). 

### 2.2. Effect of Glucose on PMN-Mediated Phagocytosis

To reveal the reason for the higher virulence of the *HGT1* knockout strain of *Candida albicans* compared to the parental strain SN152 in the murine model, further in vitro analyses were performed. It was shown previously that depletion of neutrophils causes worse outcomes of candidiasis in disseminated, oropharyngeal, and vaginal candidiasis [15,16]. It is essential to know whether *Candida* grown at different glucose concentrations alters the phagocytosis ability of polymorphonuclear leukocytes (PMNs) in addition to the dysfunction of neutrophils in hyperglycemia [17]. After growing the different strains in 0.1%, 0.2%, 0.3%, or 2% glucose, and co-incubating the serum-opsonized and FITC-labeled yeast cells with PMNs, the samples were analyzed using flow cytometry.

**Figure 2:**
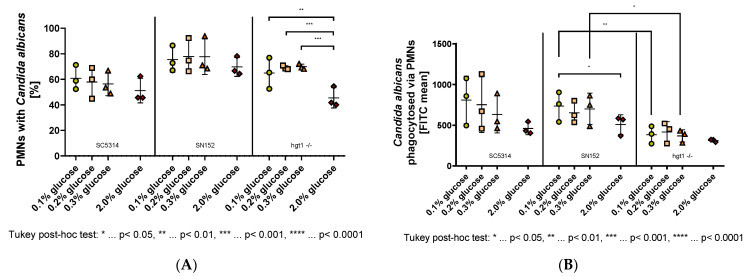
Phagocytosis of opsonized *Candida albicans* yeast cells grown at different glucose concentrations overnight by polymorphonuclear leukocytes (PMNs). Yeast cells of wild type (SC5314), parental (SN152), and *HGT1* knockout (hgt1-/-) strains were incubated in yeast peptone medium with either 0.1%, 0.2%, 0.3%, or 2% glucose, opsonized with 40% normal human serum (NHS) and labeled with fluorescein isothiocyanate (FITC). Freshly isolated PMNs were added and their membrane was labeled with AlexaFluor^®^ 647-conjugated wheat germ agglutinin (WGA). The percentage of double-positive PMNs (**A**) was measured using fluorescence-activated cell sampler (FACS) Verse with BD software and the amount of phagocytosed *Candida albicans* yeast cells is shown as FITC mean (**B**). The results shown were gained by three independently performed experiments (mean ± SD). Stars (*) indicate the level of significance for an adjusted *p*-value, comparing pairs of groups using a Tukey post-hoc analysis after the one-way ANOVA reported significance.

The results revealed that the glucose concentration in which the *Candida albicans* wild type strain SC5314 was incubated had a minor effect on both the percentage of PMNs phagocytosing yeast cells (Figure 2A left panel) and the amount of SC5314 cells phagocytosed by PMNs (Figure 2B left panel). Nevertheless, a tendency towards a lower percentage of PMNs phagocytosing yeast cells with higher glucose concentration could be seen (Figure 2A left panel). Additionally, it was shown that there was a tendency of fewer yeast cells being phagocytosed by PMNs with higher glucose concentration (Figure 2B left panel). 

The parental strain SN152, although apparently being phagocytosed by more PMNs compared to the wild type strain, showed no significant difference between the physiological glucose concentrations (Figure 2A middle panel). A tendency towards lower phagocytosis was observed when comparing these low glucose concentrations with 2% glucose (Figure 2A middle panel). Moreover, fewer SN152 yeast cells, which have previously been incubated in 2% glucose, were phagocytosed by each PMN compared to the yeast cells of the same strain incubated in 0.1% glucose (Figure 2B middle panel). The conducted one-way ANOVA revealed that this difference was significant, F(3,8) = 4.275, *p* = 0.0446. 

Regarding the amount of PMNs phagocytosing hgt1-/- yeast cells, the percentage differed between 2% glucose and each physiological glucose concentration, while it did not differ within the physiological glucose concentrations (Figure 2A right panel). These differences were shown significant by a one-way ANOVA, F(3,8) = 24.76, *p* = 0.0002. The Tukey post-hoc analysis revealed that the mean percentage of PMNs phagocytosing *Candida* cells was significantly decreased in yeast cells grown in 2% glucose compared to 0.1% glucose (−19.44, 95% CI (−29.87,−9.019), *p* = 0.0015), 0.2% glucose (−23.62, 95% CI (−34.05,−13.2), *p* = 0.0004), and 0.3% glucose (−24.4, 95% CI (−34.82,−13.98), *p* = 0.0003; Figure 2A right panel). Like SN152, the *HGT1* knockout mutant (hgt1-/-) did not show any significant difference for the amount of yeast cells phagocytosed by PMNs when incubated at physiological glucose concentrations (Figure 2B right panel). In contrast to SN152, no significant difference could be shown in FITC mean between physiological glucose concentrations and the yeast cells incubated in 2% glucose (Figure 2B right panel). However, comparing the FITC mean of hgt1-/- to SN152, fewer hgt1-/- cells were phagocytosed at all glucose concentrations (Figure 2B middle and right panel). A conducted one way ANOVA and following Tukey post-hoc test revealed that this visible difference was significant at 0.1% glucose (−350.7, 95% CI (−638.5,−62.84), *p* = 0.0084) and 0.3% glucose (−331.4, 95% CI (−619.3,−43.61), *p* = 0.0147).

### 2.3. Effect of Glucose on the Deposition of C3b/iC3b on C. albicans

C3 deficient mice of the conducted murine model exhibited the worst outcome when challenged with *Candida albicans* cells. Since iC3b acts as an opsonizing molecule and stimulates internalization by interaction with receptors on phagocytes [1], the C3b and iC3b deposition on *Candida albicans* grown at different glucose concentrations needed checking. For this the different *Candida* strains were grown in 0.1%, 0.2%, 0.3%, or 2% glucose, opsonized with human serum, adding an anti-C3b/iC3b antibody, and analyzed using flow cytometry.

**Figure 3:**
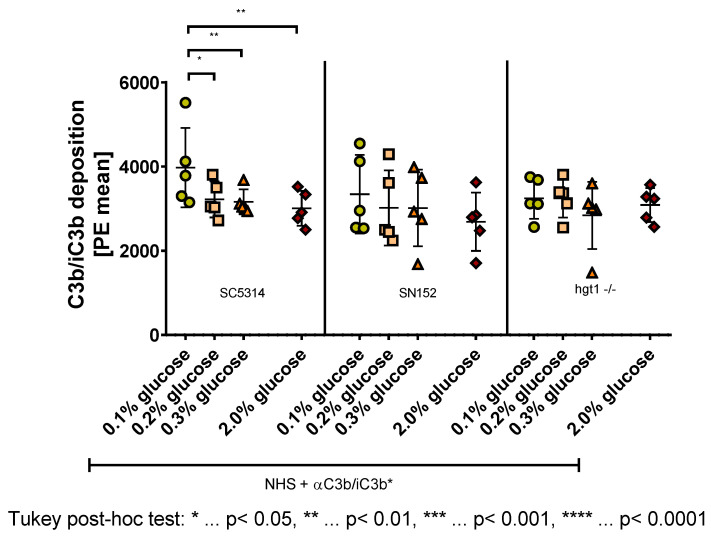
C3b/iC3b deposition on the surface of *Candida albicans* yeast cells incubated overnight at different glucose concentrations. Yeast cells of wild type (SC5314), parental (SN152), and *HGT1* knockout (hgt1-/-) strains were incubated in yeast peptone medium with either 0.1%, 0.2%, 0.3%, or 2% glucose and opsonized with 10% normal human serum (NHS). A specific anti C3b/iC3b antibody (αC3b/iC3b) was used and the deposition was measured using fluorescence-activated cell sampler (FACS) Verse with BD software. The deposition of C3b/iC3b is shown in phycoerythrin (PE) mean. The results shown were gained by five independently performed experiments (mean ± SD). Stars (*) indicate the level of significance for an adjusted *p*-value, comparing pairs of groups using a Tukey post-hoc analysis after the one-way ANOVA reported significance.

Analyzing the samples showed that the most C3b/iC3b was deposited on the surface of wild type strain SC5314, which was incubated with 0.1% glucose (M = 3973, SD = 890.9) compared with the other tested glucose concentrations and strains (Figure 3 left panel). The Tukey post-hoc analysis revealed, that within the wild type strain the cells incubated in media with 0.1% glucose overnight displayed more C3b/iC3b on their cell surface than those incubated in 0.2% glucose (753.3, 95% CI (151.7,1355), *p* = 0.0139), 0.3% glucose (812.8, 95% CI (211.2,1414), *p* = 0.0085), and 2% glucose (964.5, 95% CI (362.8,1566), *p* = 0.0026). Apart from these significant differences, a tendency of lower C3b/iC3b deposition with increasing glucose concentration on this strain was observed (Figure 3 left panel).

A conducted one-way ANOVA showed no significant difference between SN152 yeast cells grown at the different glucose concentrations. Nevertheless, a tendency for lower C3b/iC3b deposition on the cell surface with increasing glucose concentration was visible (Figure 3 middle panel).

In contrast to SC5314 and SN152 yeast cells, for the *HGT1* knockout strain (hgt1-/-), no difference or tendency in C3b/iC3b deposition on the cell surface was detected (Figure 3 right panel).

### 2.4. Effect of Glucose on the Deposition of FH on C. albicans

Factor H (FH) is used by *Candida albicans* to avoid detrimental effects from complement [18]. To enlighten the effect of glucose on the deposition of this protein on the surface of *Candida* cells, an in vitro experiment was conducted with yeast cells incubated overnight in either 0.1%, 0.2%, 0.3%, or 2% glucose and opsonized with human serum. To detect the amount of FH on the surface of these cells, an anti-FH antibody was added and flow cytometry was used to measure the samples.

**Figure 4:**
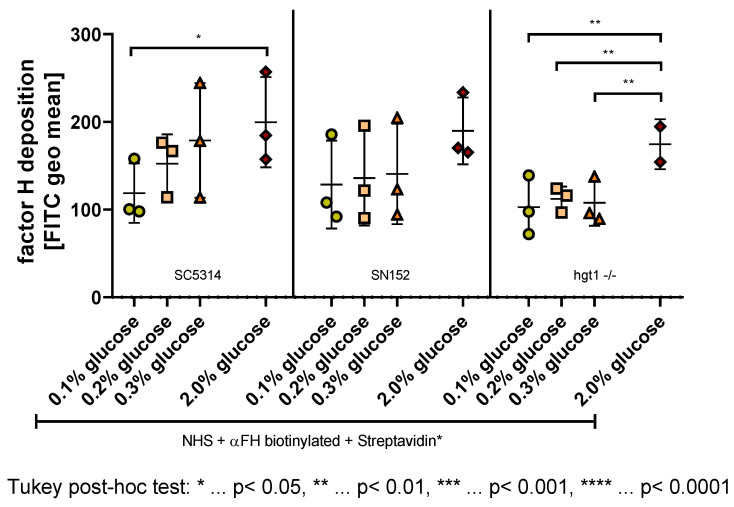
Factor H (FH) deposition on the surface of *Candida albicans* yeast cells incubated overnight at different glucose concentrations. Yeast cells of wild type (SC5314), parental (SN152), and *HGT1* knockout (hgt1-/-) strains were incubated in yeast peptone medium with either 0.1%, 0.2%, 0.3%, or 2% glucose and opsonized with 40% normal human serum (NHS). A specific anti-FH antibody (αFH) was used and the deposition was measured using fluorescence-activated cell sampler (FACS) Verse with BD software. The deposition of FH is shown in fluorescein isothiocyanate (FITC) geometric mean (geo mean). The results shown were gained by three independently performed experiments (mean ± SD). Stars (*) indicate the level of significance for an adjusted *p*-value, comparing pairs of groups using a Tukey post-hoc analysis after the one-way ANOVA reported significance.

Regarding the wild type strain SC5314, a tendency for higher FH deposition with higher glucose concentration was observed (Figure 4 left panel). Tukey post-hoc analysis revealed that on SC5314 yeast cells incubated in 2% glucose, significantly more FH is binding than on yeast cells of the same strain incubated in 0.1% glucose (80.89, 95% CI (9.219,152.6), *p* = 0.0282) (Figure 4 left panel), which correlates negatively with the C3b/iC3b deposition on this strain (Figure 3 left panel).

This negative correlation with the C3b/iC3b deposition was also detected as a visible, albeit not significant, tendency in the parental strain SN152 (Figure 4 middle panel; Figure 3 middle panel).

Unlike the yeast cells of SC5314 and SN152, the *HGT1* knockout mutant hgt1-/- did not show any differences in FH deposition when comparing the physiological glucose concentrations (Figure 4 right panel), which correlates with the C3b/iC3b results described above (Figure 3 right panel). In contrast to the C3b/iC3b deposition (Figure 3 right panel), the FH deposition on hgt1-/- yeast cells grown in 2% glucose did however differ from the physiological glucose concentrations (Figure 4 right panel). A conducted Tukey post-hoc analysis showed that significantly less FH is binding to the cell surface of hgt1-/- grown in 0.1% (−71.61, 95% CI (−112.4,−30.77), *p* = 0.0022), 0.2% (−62.28, 95% CI (−103.1,−21.44), *p* = 0.0053), and 0.3% glucose (−66.83, 95% CI (−107.7,−26.00), *p* = 0.0035).

## 3. Discussion

Understanding the mechanisms behind the pathogenesis of candidiasis is crucial to finding new approaches to treat patients suffering from this disease, accounting for an estimated 10,000 deaths per year in Europe [19]. Hence, we here analyzed the relevance of the complement system, as an essential part of the innate immunity, and glucose on the pathogenesis of *Candida albicans*. We investigated three aspects of the interplay of *Candida albicans*, the complement system, and glucose. Firstly, we studied in vitro the effect of glucose on the phagocytosis of three different *Candida albicans* strains grown in 0.1%, 0.2%, 0.3%, or 2% glucose and opsonized with human serum by granulocytes. We used the following strains: a wild type strain (SC5314), a high-affinity glucose transporter 1 knockout strain (hgt1-/-), which is lacking Hgt1, a factor H-binding molecule known for a glucose-dependent expression, and its parental strain (SN152). Patients suffering from diabetes mellitus experience several modulations of cellular and humoral defenses including the complement system [20]. With respect to cellular defenses, hyperglycemia particularly affects the functions of neutrophils, essential for eliminating pathogens. High glucose concentrations impede phagocytosis of lipopolysaccharide-coated particles [21], opsonized Gram-negative and Gram-positive bacteria [22], and *Staphylococcus aureus* [23]. In this study, we showed that the phagocytosis of *Candida albicans* SC5341 and SN152 is also decreased at higher glucose concentrations. For the *HGT1* knockout mutant (hgt1-/-), there was no difference in phagocytosis detected within physiological glucose concentrations (0.1–0.3%). Nevertheless, in unphysiological high 2% glucose, the percentage of granulocytes phagocytosing hgt1-/- decreased compared to physiological glucose concentrations, while the amount of phagocytosed cells did not differ. However, the differences in phagocytosis were weak and much less pronounced than expected.

Secondly, we checked whether glucose has an effect on the yeast cell surface of *Candida albicans* altering in particular the C3b/iC3b deposition and FH deposition. Besides the immune system being altered by hyperglycemia, it was also shown that high glucose concentrations lead to modifications on the pathogen side such as increased adhesion, invasion [24], and stress resistance [25] of *Candida albicans*. The in vitro experiments performed in this study showed an additional alteration of *Candida albicans* strains by glucose. The incubation of *Candida albicans* SC5314 and SN152 at different glucose concentrations led to a lower C3b/iC3b deposition with increasing glucose concentration. It is known that glucose binds to the biochemically active site of C3 at high glucose concentrations in a nonenzymatic manner, changing the tertiary structure of the molecule, inhibiting the activation of C3b by *Staphylococcus aureus*, and the deposition of C3b and iC3b on *Staphylococcus aureus* [26,27]. Since we wanted to investigate the role of glucose on *Candida albicans* alone, we circumvented this effect of glucose on the complement by executing several washing steps and therefore removing residual glucose before the opsonization with normal human serum. Besides the altered C3b/iC3b deposition, we also showed higher FH deposition with increasing glucose concentrations.

However, as indicated before, it must be stated that the effects of the glucose concentration in vitro were much less pronounced than expected.

Further investigations are needed to clarify the mechanisms behind the glucose-dependent alterations of *Candida albicans* that might interfere with the complement deposition.

When growing in a host, pathogens and commensals alike have to face the innate immunity. To avoid recognition or destruction by the immune system, microorganisms have evolved several immune evasion mechanisms. Especially the complement system as a crucial bridge between the innate and the adaptive immunity is often the target for these evasion mechanisms. One of these is to bind factor H (FH), which is the major soluble regulator of the alternative pathway, to their surface [28]. We here showed, as mentioned above, that FH binding in *Candida albicans* is glucose-dependent. We revealed that knocking out *HGT1* diminishes this glucose dependency of FH, except at 2% glucose. Besides Hgt1, the expression of another FH-binding molecule of *Candida albicans* is linked to glucose, namely Gpd2. While the gene expression of *GPD2* is up-regulated at higher glucose concentrations [25], the gene expression of *HGT1* was found to be significantly lower in high glucose compared to euglycemic glucose levels [14]. This up-regulation of Gpd2 might be responsible for the increased FH deposition on hgt1-/- incubated at 2% glucose.

Thirdly, we examined whether the absence of Hgt1 or C3 can be linked to the survival in a murine model. We therefore intravenously challenged euglycemic, diabetic, and C3-deficient mice with SN152 and in addition euglycemic and diabetic mice with hgt1-/-. Survival and clinical status were monitored over 14 days. Although it was shown previously that *Candida albicans* can escape mouse neutrophils unlike human neutrophils by outgrowing as hyphae [29], the lethality in euglycemic mice was low in our study. As demonstrated in several other studies [30,31,32], the outcome of a systemic *Candida albicans* infection was worse in diabetic animals, which can be explained by altered cellular and humoral defenses in hyperglycemia as elaborated above [20]. Furthermore, *Candida albicans* exhibits an increased resistance towards oxidative and cationic stress at higher glucose concentrations [25]. These stress responses might lead to better protection against the antimicrobial properties of phagocytes, which rely either on oxidative burst [33] or the influx of cations [34]. Additionally, we could show the crucial part of C3 in defense against *Candida albicans* SN152 in immunosuppressed animals. This is supported by the findings of Tsoni and coworkers regarding the wild type strain SC5314, which exhibited a higher virulence in C3 deficient animals [35]. When conducting our study, knocking out C3 was thought to result in lacking complement opsonization, but the formation of the membrane attack complex (MAC) could still work due to thrombin cleaving C5 into C5a and C5b [36]. It was revealed recently that a conformational change of C5 is needed for this thrombin-mediated cleavage and is therefore probably not happening in vivo or solely under certain circumstances [37]. We hypothesize that other components could be able to cleave C5 due to a similar inflammation reaction shown by Tsoni [35].

**Figure 5:**
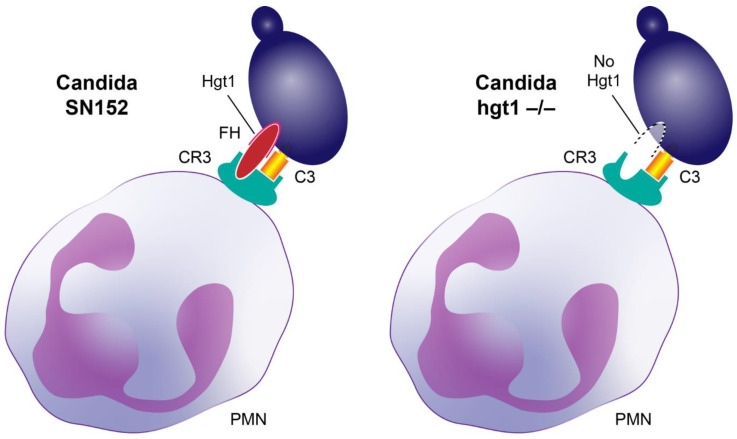
Factor H (FH) bridging *Candida albicans* cells to professional phagocytes. Both activated C3 (hatched yellow box) and FH (red ellipse) bind to complement receptor 3 (CR3, cyan receptor). Upon opsonization, activated C3 is deposited on *Candida albicans* (violet cell) and FH binds to its receptor on the yeast (such as Hgt1). Left: binding of *Candida albicans* parental strain (SN152) to the phagocyte CR3 via both FH and activated C3. Right: Proposed model of an assumed less efficient binding of *Candida albicans* hgt1-/- mutant lacking an important FH-binding molecule (indicated by dotted line) via just activated C3 to the phagocyte CR3 (in vivo some FH molecules will obviously bind, but with lower affinity).

When knocking out *HGT1*, a complement evasion molecule, the resulting hgt1-/- strain should have shown a reduced virulence in a murine model due to reduced FH deposition and the subsequent exerted functions by this major regulator protein of the alternative pathway [9]. In contrast to this hypothesis, the murine model revealed that knocking out *HGT1* in *Candida albicans* increased the virulence of the resulting hgt1-/- strain in euglycemic mice. In addition, in diabetic mice SN152 showed a comparably high virulence, which might be due to the downregulation of Hgt1 in this strain at higher glucose concentrations shown in our previous work [14]. Furthermore, the reduced FH deposition on *Candida albicans* hgt1-/- incubated at physiological glucose concentrations did not lead to a markedly higher phagocytosis. On the contrary, fewer hgt1-/- incubated at physiological glucose concentrations were phagocytosed compared to SN152. This could be explained by a different property of FH: besides regulating the complement system by inactivating C3b and accelerating the decay of the C3- and C5-convertase of the alternative pathway [9], it binds complement receptor 3 (CR3, CD11b/CD18) [38]. CR3, a receptor, is inter alia involved in phagocytosis and mainly expressed on macrophages, monocytes, granulocytes, and natural killer cells. *Candida*-bound FH interacts with CR3, therefore bridging the pathogen to professional phagocytes like PMNs and supporting the clearance of *Candida albicans* [38,39] (Figure 5).

Confirming the relevance of CR3 in *Candida* infections, mice deficient in CR3 presented with an aggravating course of infection compared to age-matched control mice [40]. The strongest support for our murine model is the findings of Soloviev and coworkers, who monitored the virulence of *Candida albicans* Pra1 deletion mutant compared to a CAI-12 as a control strain and gained similar results to those of our murine model, namely that the elimination of the FH-binding molecule leads to a higher virulence of the mutant strain [40]. FH bridging the pathogen to phagocytes can explain the hgt1-/- mutant results mentioned above.

To elucidate whether the induction of FH-binding molecules such as Pra1 or Hgt1 may represent a new therapeutic approach to tackle *Candida* infections, a mutant overexpressing Pra1 or Hgt1 has to be generated. Such mutant(s) can be used to compare the phagocytosis in vitro with the parental strain and, if proven promising, translated into a murine model.

Regarding the media, which are normally used to grow *Candida albicans*, these mostly contain 2% glucose like YPD, yeast nitrogen base (YNB), and synthetic low ammonium dextrose (SLAD) [41]. Alternative media for *Candida albicans*, which should be used if the results should depict physiological conditions, would be Medium 199, serum agar medium, spider medium, and Lee’s medium containing either 0.1% or 0.24% glucose [41]. These glucose concentrations are those of euglycemic or prediabetic patients [11]. The same issue can be observed with antifungal susceptibility testing (AFST) methods, which are performed using high glucose concentrations to ensure fungal growth. When working according to the EUCAST guidelines, RPMI with 2% glucose is needed for the broth microdilution method [42,43]; according to the CLSI guidelines, either 0.2% glucose is used for the broth microdilution method or 2% glucose for the agar diffusion method [43,44], and the agar plates for the agar diffusion Etest method contain 2% glucose [43]. The difference in glucose concentrations used in these methods could be partially responsible for the differences in the minimum inhibitory concentrations (MICs) when testing the same strains using different methods [43], and thus physiological glucose concentration should always be considered.

## 4. Materials and Methods

### 4.1. Reagents and Media

Streptozotocin, Histopaque-1077, RPMI 1640 with L-glutamine, without glucose and sodium bicarbonate, sodium phosphate dibasic (Na_2_HPO_4_), fetal calf serum (FCS), bovine serum albumin (BSA), ethylene glycol-bis (β-aminoethyl ether) tetraacetic acid (EGTA), t-Octylphenoxypolyethoxyethanol (Triton X-100), trypan blue solution 0.4%, and fluorescein isothiocyanate (FITC) isomer I were purchased from Sigma Aldrich (St. Louis, MO, USA). Sodium hydrogen bicarbonate (NaHCO_3_), sodium dihydrogen phosphate dihydrate (NaH_2_PO_4_ × 2H_2_O), yeast extract, and Tween 20 were obtained from MERCK (Darmstadt, Germany). D (+)-glucose, peptone from soy, agar, sodium chloride (NaCl), calcium chloride (CaCl_2_), magnesium chloride hexahydrate (MgCl_2_ × 6H_2_O), ethylenediaminetetraacetic acid (EDTA), formaldehyde solution 37%, and sodium carbonate anhydrous (Na_2_CO_3_) were bought from Carl Roth (Karlsruhe, Germany). Fluorescein isothiocyanate (FITC)-conjugated streptavidin was ordered from Invitrogen AG (Carlsbad, CA, USA). Phycoerythrin (PE)-conjugated anti-human C3b/iC3b antibody and biotinylated mouse anti-human factor H (FH) were supplied by BioLegend (San Diego, CA, USA). Alexa Fluor^®^ 647—conjugated wheat germ agglutinin (WGA) was purchased from Life Technologies (Carlsbad, CA, USA).

### 4.2. Mice

C57Bl/6J mice were purchased from Charles River Laboratories (Sulzfeld, Germany) and used as controls (wild type mice; wt) or 130 µg/g streptozotocin, which is toxic for the insulin-producing beta cells of the pancreas, was administered intraperitoneally to obtain diabetic mice. C3 deficient (ΔC3) mice are genetically modified C57Bl/6 mice and bred at the Institute of Hygiene and Medical Microbiology in Innsbruck. The murine experiments were conducted in conformity with national law and guidelines of the European Communities and performed at the Institute of Hygiene and Medical Microbiology in Innsbruck. The mice were housed at a 12-h alternating light–dark cycle and provided with standardized feed and sterilized water *ad libitum*. Five percent saccharose was added to the sterilized water of diabetic mice.

### 4.3. Candida albicans Strains

The *Candida albicans* strains used were SC5314 (a wild type genotype) as control strain and SN152 (a strain auxotrophic for arginine, leucine, and histidine) as parental strain. The latter was used for knocking out *HGT1* (hgt1-/-) according to standard protocols [45]. The mutant strain was generated previously by Lesiak-Markowicz and colleagues [7]. The strains were routinely maintained on YPD agar (yeast extract 1%, peptone from soy 2%, D (+)-glucose 2%, and agar 1.5%) plates. For the FH and C3b/iC3b deposition on *Candida albicans* surface and the PMN mediated fungal killing, an overnight (O/N) culture of each strain was grown at 30 °C and under gentle shaking in YP-medium containing 0.1%, 0.2%, 0.3%, or 2% glucose. For the murine model of disseminated candidiasis, an O/N culture of SN152 and hgt1-/- was produced in YP-medium containing 0.1%, 0.4%, or 2% glucose, incubated at 30 °C and under gentle shaking.

### 4.4. Murine Model of Disseminated Systemic Candidiasis

For the model of disseminated *Candida* infection, groups of seven to twelve 7-week-old mice were infected intravenously with yeast cells of either *Candida albicans* SN152 or hgt1-/- strain (6 × 10^2^/g mouse in 100 µL 0.9% NaCl). *Candida* strains used for the infection of euglycemic (wt) mice were grown in YP-medium containing 0.1% glucose overnight before inoculation. For ΔC3 mice, an overnight culture of the different *Candida* strains grown in YP-medium containing 2% glucose was used, and for diabetic animals, the strains were grown in YP-medium containing 0.4% glucose before inoculation. All mice were immunosuppressed starting at day −4 before infection with 50 µg/g cyclophosphamide. This medication was attributed every second day to maintain the immunosuppression. To induce diabetes 130 µg/g streptozotocin was administered intraperitoneally at day −10 prior to infection. Furthermore, 5% saccharose was added to the distilled drinking water from day −9 forward and blood sugar was controlled before the infection at day −6 and day −3. If the blood sugar level was too low, additional streptozotocin was administered, depending on the measured blood sugar. One hundred micrograms per gram of streptozotocin was administered at blood glucose levels of <10 mmol/L, 70 µg/g at 10–12.5 mmol/L, and 40 µg/g at 12.5–15 mmol/L. After infection, the sugar levels in blood and urine were measured regularly. Body weight and temperature were determined twice a day. A clinical assessment was performed at least twice a day after the infection. Blood and urine samples were obtained on day 1, day 8, and the day of exitus, which was either day 14 post-infection or when the animals met humane endpoints [46] as defined in the animal proposal, such as loss of body weight, loss of mean body temperature, missing intake of water or food, and appearance of neurological symptoms. On the day of exitus, animals were euthanized by cervical dislocation under isoflurane anesthesia [47]. The Federal Ministry of Education, Science and Research, Republic of Austria approved the conducted animal experiments (BMWFW-66.011/0196-WF/V/3b/2016, BMBWF-66.011/0102-V/3b/2018).

### 4.5. Isolation of Polymorphonuclear Leukocytes (PMNs)

Fresh blood was obtained from informed consent healthy human donors, approved by the Ethics Committee of the Medical University of Innsbruck, Austria (ECS1166/2018, 14 November 2018). A density gradient centrifugation was performed by adding Histopaque-1077 and RPMI medium to the EDTA-blood and centrifuging it at 570× *g* for 25 min without break. The supernatant was discarded until 5 mm above the separation line. The PMNs were situated at the bottom mixed with erythrocytes. To remove surplus Histopaque-1077, the liquid was washed with 1x PBS and centrifuged with break at 500× *g* for 10 min. The supernatant was discarded and hypotonic erythrocyte lysis was performed by adding distilled water to the cell pellet for 20 s. The physiological osmolarity was restored by the addition of the same amount of 2× PBS. The suspension was centrifuged at 500× *g* for 5 min and the supernatant was removed. Lysis and centrifugation were repeated until the pellet appeared white. The supernatant was discarded and the pellet containing PMNs was resuspended in RPMI medium containing 0.1% glucose. Purity was determined by light microscopy.

### 4.6. PMN-Mediated Phagocytosis of Candida albicans Ex Vivo

The O/N culture of SC5314, SN152, and hgt1-/- was washed two times with 5 mL 1× PBS and centrifuged at 2000× *g* for 5 min. The supernatant was discarded and the pellet was resuspended in 1 mL 1× PBS. *Candida* yeast cells were opsonized for 30 min at 37 °C and 5% CO_2_ with 40% fresh normal human serum (NHS) from an NHS pool and 20% 1× PBS++ (0.15 mM CaCl_2_ and 1 mM MgCl_2_ were added to 1× PBS) was added. As a control, the NHS was replaced with heat-inactivated NHS, which was achieved by heating the NHS for 30 min at 60 °C in a water bath. After the incubation period, the yeast cells were washed two times with 1 mL 1× PBS and centrifuged for 5 min at 2000× *g*. The supernatant was discarded and *Candida* was resuspended in 1 mL 1× PBS. One milliliter of staining solution, consisting of 9.5 mL 0.1 M Na_2_CO_3_, 0.5 mL 1× PBS/0.1% Tween 20 and 1 mg FITC, was added and the cells were incubated for 15 min at 37 °C. To separate *Candida* from unbound FITC, the suspension was centrifuged at 900× *g* for 6 min, the supernatant discarded, and washed two times with 1× PBS. After the washing steps, the *Candida* was resuspended in 1 mL 1× PBS.

Isolated PMNs were diluted using RPMI with different glucose concentrations, namely 0.1%, 0.2%, 0.3%, and 2%. A concentration of 1 × 10^5^ PMNs were added to 5 mL round-bottom tubes containing 1 × 10^6^ FITC-labeled *Candida* and 100 µL RPMI/10% FCS_HI_ (heat-inactivated fetal calf serum). For phagocytosis, the 5 mL round-bottom tubes were incubated 1 h at 37 °C and 5% CO_2_, under gently shaking. Thirty minutes before the incubation period ended, 0.5 µg WGA Alexa Fluor 647 was used to mark the PMNs. Phagocytosis was stopped by adding 200 µL 1× PBS/1% formalin per 5 mL round-bottom tube and incubating them for 30 min at room temperature. The samples and controls were washed with 300 µL 1× PBS, centrifuged at 200× *g* for 7 min, and resuspended in 100 µL 1× PBS/1% formalin, fixed for 30 min at room temperature, and afterwards, the tubes were covered with parafilm.

The PMN-mediated phagocytosis of labeled yeast cells was measured using the fluorescence-activated cell sampler (FACS) Verse and BD software (BD Bioscience, San Diego, CA, USA). WGA-positive PMNs were gated by APC-A vs. FSC-A color density plot and 10,000 gate events were analyzed. The WGA-positive gate was further investigated for FITC-positivity using a color density plot FITC-A vs. FSC-A.

### 4.7. FH and C3b/iC3b Deposition on C. albicans Surface by Fluorescence-Activated Cell Sampling (FACS)

The O/N culture of SC5314, SN152, and hgt1-/- was washed as described above. One million *Candida* yeast cells were opsonized for 10 min at 37 °C and 5% CO_2_ with 40% NHS from an NHS pool for FH deposition and 10% NHS for C3b/iC3b deposition, respectively. Fifty percent (v/v_NHS_) 1× PBS++ was added to supply a sufficient amount of ions necessary for the activation of the complement system. As negative controls, the NHS was either replaced with 1× PBS or with heat-inactivated NHS. Further controls were achieved by replacing 1× PBS ++ with either 1× PBS/0.01 M EDTA, which blocks the activation of all complement pathways, or with 1× PBS/0.01 M EGTA/0.0025 M MgCl_2_, blocking classical pathway activation. The opsonized cells and controls were washed two times with 300 µL 1× PBS, centrifuged at 3000× *g* for 5 min and the supernatant was discarded. Nonspecific binding sites were blocked with 200 µL 1× PBS/0.1% BSA for 30 min at room temperature, under gentle shaking. Eight microliters biotinylated mouse anti-human FH antibody (0.6 mg/mL) was directly added to each tube to detect FH deposition or 5 µL PE-conjugated mouse anti-human C3b/iC3b (0.1 mg/mL) for C3 deposition. The samples were incubated at room temperature for 60 min. After the incubation period, the samples were washed with 300 µL 1× PBS and centrifuged at 3000× *g* for 5 min. The supernatant was discarded and the tubes for the C3b/iC3b deposition were resuspended in 300 µL 1× PBS/1% formalin, fixed for 30 min at room temperature, and afterwards the test tubes were covered with parafilm to avoid evaporation. For the FH deposition, the pellet was resuspended with FITC-conjugated streptavidin diluted 1:50 in 200 µL 1× PBS. These samples were incubated at room temperature for 60 min. The tubes were washed with 300 µL 1x PBS, centrifuged at 3000× *g* for 5 min, and resuspended in 300 µL 1× PBS/1% formalin, fixed for 30 min at room temperature, and afterwards, the tubes were covered with parafilm.

The deposition of FH and C3b/iC3b were measured independently using the FACS Verse and BD software (BD Bioscience, San Diego, CA, USA). The yeast cells were gated by a SSC-A vs. FSC-A density plot. Ten thousand gate events were analyzed. Single yeast cells were furthermore gated by FSC-H vs. FSC-W. The positive population was detected using a one-color density plot SSC-A vs. FITC-A for anti-FH and SSC-A vs. PE-A for anti-C3b/iC3b.

### 4.8. Statistical Analyses

Data obtained were analyzed and plotted using GraphPad Prism 8. In vitro experiments were performed 3 to 6 times on different days. PMNs were isolated from different healthy donors. To show significant differences, one-way ANOVAs were performed and Tukey post-hoc analyses were used for further analyzing differences between the different groups. The survival curves were analyzed using the Mantel–Cox test. The reported *p*-values throughout the manuscript refer to adjusted *p*-values. The threshold for statistical significance was <0.05.

## Data Availability

Not applicable.
